# The impact of the metabotropic glutamate receptor and other gene family interaction networks on autism

**DOI:** 10.1038/ncomms5074

**Published:** 2014-06-13

**Authors:** Dexter Hadley, Zhi-liang Wu, Charlly Kao, Akshata Kini, Alisha Mohamed-Hadley, Kelly Thomas, Lyam Vazquez, Haijun Qiu, Frank Mentch, Renata Pellegrino, Cecilia Kim, John Connolly, AGP Consortium, Dalila Pinto, Alison Merikangas, Lambertus Klei, Jacob A.S. Vorstman, Ann Thompson, Regina Regan, Alistair T. Pagnamenta, Bárbara Oliveira, Tiago R. Magalhaes, John Gilbert, Eftichia Duketis, Maretha V. De Jonge, Michael Cuccaro, Catarina T. Correia, Judith Conroy, Inês C. Conceição, Andreas G. Chiocchetti, Jillian P. Casey, Nadia Bolshakova, Elena Bacchelli, Richard Anney, Lonnie Zwaigenbaum, Kerstin Wittemeyer, Simon Wallace, Herman van Engeland, Latha Soorya, Bernadette Rogé, Wendy Roberts, Fritz Poustka, Susana Mouga, Nancy Minshew, Susan G. McGrew, Catherine Lord, Marion Leboyer, Ann S. Le Couteur, Alexander Kolevzon, Suma Jacob, Stephen Guter, Jonathan Green, Andrew Green, Christopher Gillberg, Bridget A. Fernandez, Frederico Duque, Richard Delorme, Geraldine Dawson, Cátia Café, Sean Brennan, Thomas Bourgeron, Patrick F. Bolton, Sven Bölte, Raphael Bernier, Gillian Baird, Anthony J. Bailey, Evdokia Anagnostou, Joana Almeida, Ellen M. Wijsman, Veronica J. Vieland, Astrid M. Vicente, Gerard D. Schellenberg, Margaret Pericak-Vance, Andrew D. Paterson, Jeremy R. Parr, Guiomar Oliveira, Joana Almeida, Cátia Café, Susana Mouga, Catarina Correia, John I. Nurnberger, Anthony P. Monaco, Elena Maestrini, Sabine M. Klauck, Hakon Hakonarson, Jonathan L. Haines, Daniel H. Geschwind, Christine M. Freitag, Susan E. Folstein, Sean Ennis, Hilary Coon, Agatino Battaglia, Peter Szatmari, James S. Sutcliffe, Joachim Hallmayer, Michael Gill, Edwin H. Cook, Joseph D. Buxbaum, Bernie Devlin, Louise Gallagher, Catalina Betancur, Stephen W. Scherer, Joseph Glessner, Hakon Hakonarson

**Affiliations:** 1 The Center for Applied Genomics, The Children’s Hospital of Philadelphia, Philadelphia, Pennsylvania 19104, USA; 2 Department of Pediatrics, University of Pennsylvania School of Medicine, Philadelphia, Pennsylvania 19104, USA; 83 List of participants and their affiliations appear at the end of the paper.; 3 Seaver Autism Center for Research and Treatment, Icahn School of Medicine at Mount Sinai, New York, New York 10029, USA;; 4 Department of Psychiatry, Icahn School of Medicine at Mount Sinai, New York, New York 10029, USA;; 5 Department of Genetics and Genomic Sciences, Icahn School of Medicine at Mount Sinai, New York, New York 10029, USA;; 6 The Mindich Child Health and Development Institute, Icahn School of Medicine at Mount Sinai, New York, New York 10029, USA;; 7 The Icahn Institute for Genomics and Multiscale Biology, Icahn School of Medicine at Mount Sinai, New York, New York 10029, USA;; 8 Friedman Brain Institute, Icahn School of Medicine at Mount Sinai, New York, New York 10029, USA;; 9 INSERM, U1130, 75005 Paris, France;; 10 CNRS, UMR 8246, 75005 Paris, France;; 11 Sorbonne Universités, UPMC Université Paris 6, Neuroscience Paris Seine, 75005 Paris, France;; 12 Program in Genetics and Genome Biology, The Centre for Applied Genomics, The Hospital for Sick Children, Toronto, Ontario, Canada M5G1L7;; 13 Discipline of Psychiatry, School of Medicine, Trinity College Dublin, Dublin 8, Ireland;; 14 Department of Psychiatry, University of Pittsburgh School of Medicine, Pittsburgh, Pennsylvania 15213, USA;; 15 Department of Psychiatry, Brain Center Rudolf Magnus, University Medical Center Utrecht, 3584CX Utrecht, The Netherlands;; 16 Department of Psychiatry and Behavioural Neurosciences, Offord Centre for Child Studies, McMaster University, Hamilton, Ontario, Canada L8S 4K1;; 17 National Children's Research Centre, Our Lady's Children's Hospital, Dublin 12, Ireland;; 18 Academic Centre on Rare Diseases, School of Medicine and Medical Science, University College Dublin, Dublin 4, Ireland;; 19 Wellcome Trust Centre for Human Genetics, University of Oxford, Oxford OX3 7BN, UK;; 20 Instituto Nacional de Saúde Doutor Ricardo Jorge, 1649-016 Lisboa, Portugal;; 21 Center for Biodiversity, Functional & Integrative Genomics, Faculty of Sciences, University of Lisbon, 1749-016 Lisboa, Portugal;; 22 McLaughlin Centre, University of Toronto, Toronto, Ontario, Canada M5S1A1;; 23 Department of Neurology and Center for Autism Research and Treatment, Semel Institute, David Geffen School of Medicine, University of California, Los Angeles, California 90095, USA;; 24 John P. Hussman Institute for Human Genomics, Dr John T. Macdonald Foundation Department of Human Genetics, University of Miami School of Medicine, Miami, Florida 33136, USA;; 25 Department of Child and Adolescent Psychiatry, Psychosomatics and Psychotherapy, Goethe-University, 60528 Frankfurt am Main, Germany;; 26 Pathology and Laboratory Medicine, Perelman School of Medicine, University of Pennsylvania, Philadelphia, Pennsylvania 19104, USA;; 27 Vanderbilt Brain Institute, Center for Human Genetics Research and Department of Molecular Physiology & Biophysics, Vanderbilt University, Nashville, Tennessee 37232, USA;; 28 Children's University Hospital Temple Street, Dublin 1, Ireland;; 29 Department of Pharmacy and Biotechnology, University of Bologna, 40126 Bologna, Italy;; 30 Department of Pediatrics, University of Alberta, Edmonton, Alberta, Canada T6B 2H3;; 31 School of Education, University of Birmingham, Birmingham B15 2TT, UK;; 32 Department of Psychiatry, University of Oxford, Warneford Hospital, Oxford OX3 7JX, UK;; 33 Unité de Recherche Interdisciplinaire Octogone, Centre d'Etudes et de Recherches en Psychopathologie, Toulouse 2 University, 31058 Toulouse, France;; 34 Autism Research Unit, The Hospital for Sick Children, Toronto, Ontario, Canada M5G 1X8;; 35 Unidade de Neurodesenvolvimento e Autismo do Serviço do Centro de Desenvolvimento da Criança and Centro de Investigação e Formação Clinica, Pediatric Hospital, Centro Hospitalar e Universitário de Coimbra, 3000-602 Coimbra, Portugal;; 36 University Clinic of Pediatrics and Institute for Biomedical Imaging and Life Science, Faculty of Medicine, University of Coimbra, 3000-354 Coimbra, Portugal;; 37 Department of Pediatrics, Vanderbilt University, Nashville, Tennessee 37232, USA;; 38 Weill Cornell Medical College/New York Presbyterian Hospital Teachers College, New York, New York 10065, USA;; 39 FondaMental Foundation, 94010 Créteil, France;; 40 INSERM U955, Psychiatrie Génétique, 94010 Créteil, France;; 41 Université Paris Est, Faculté de Médecine, 94010 Créteil, France;; 42 Assistance Publique-Hôpitaux de Paris, Henri Mondor-Albert Chenevier Hospital, Department of Psychiatry, 94010 Créteil, France;; 43 Institute of Health and Society, Newcastle University, Newcastle upon Tyne NE1 4LP, UK;; 44 Institute for Juvenile Research and Department of Psychiatry, University of Illinois at Chicago, Chicago, Illinois 60608, USA;; 45 Present address: Institute of Translational Neuroscience, Department of Psychiatry, University of Minnesota, Minneapolis, Minnesota 55455, USA;; 46 Institute of Brain, Behaviour and Mental Health, University of Manchester, Manchester M139PL, UK;; 47 Manchester Academic Health Sciences Centre, Manchester M13 9NT, UK;; 48 National Centre for Medical Genetics, Our Lady's Children's Hospital, Dublin 12, Ireland;; 49 Gillberg Neuropsychiatry Centre, University of Gothenburg, 41119 Gothenburg, Sweden;; 50 Discipline of Genetics, Faculty of Medicine, Memorial University of Newfoundland, St John's, Newfoundland, Canada A1B 3V6;; 51 Institut Pasteur, Human Genetics and Cognitive Functions Unit, 75015 Paris, France;; 52 CNRS URA 2182 Genes, Synapses and Cognition, Institut Pasteur, 75015 Paris, France;; 53 Assistance Publique-Hôpitaux de Paris, Robert Debré Hospital, Department of Child and Adolescent Psychiatry, 75019 Paris, France;; 54 Department of Psychiatry and Behavioral Sciences, Duke University School of Medicine, Durham, North Carolina 27710, USA;; 55 University Paris Diderot, Sorbonne Paris Cité, 75013 Paris, France;; 56 Kings College London, Institute of Psychiatry, London SE5 8AF, UK;; 57 South London & Maudsley Biomedical Research Centre for Mental Health, London SE5 8AF, UK;; 58 Department of Psychiatry and Behavioral Sciences, University of Washington, Seattle, Washington 98195, USA;; 59 Paediatric Neurodisability, King's Health Partners, King's College London, LondonWC2R 2LS, UK;; 60 Bloorview Research Institute, University of Toronto, Toronto, Ontario, Canada M4G 1R8;; 61 Division of Medical Genetics, Department of Medicine, University of Washington, Seattle, Washington 98195-9460, USA;; 62 Department of Biostatistics, University of Washington, Seattle, Washington 98195-9460, USA;; 63 Battelle Center for Mathematical Medicine, The Research Institute at Nationwide Children's Hospital, Columbus, Ohio 43205, USA;; 64 Dalla Lana School of Public Health, Toronto, Ontario, Canada M5T 3M7;; 65 Institute of Neuroscience, Newcastle University, Newcastle upon Tyne NE2 4HH, UK;; 66 Institute of Psychiatric Research, Department of Psychiatry, Indiana University School of Medicine, Indianapolis, Indiana 46202, USA;; 67 Department of Medical and Molecular Genetics and Program in Medical Neuroscience, Indiana University School of Medicine, Indianapolis, Indiana 46202, USA;; 68 Division of Molecular Genome Analysis, German Cancer Research Center (DKFZ), 69120 Heidelberg, Germany;; 69 Center for Applied Genomics, The Children's Hospital of Philadelphia, Philadelphia, Pennsylvania 19104, USA;; 70 Department of Pediatrics, The Perelman School of Medicine, University of Pennsylvania, Philadelphia, Pennsylvania 19104, USA;; 71 Department of Psychiatry, Division of Child and Adolescent Psychiatry, University of Miami Miller School of Medicine, Miami, Florida 33136, USA;; 72 Utah Autism Research Program, University of Utah Psychiatry Department, Salt Lake City, Utah 84108, USA;; 73 Stella Maris Clinical Research Institute for Child and Adolescent Neuropsychiatry, 56128 Calambrone, Pisa, Italy;; 74 Stanford University Medical School, Department of Psychiatry, Stanford, California 94305, USA;; 75 Department of Neuroscience, Icahn School of Medicine at Mount Sinai, New York, New York 10029, USA;; 76 Present address: Department of Psychiatry, Rush University Medical Center, Chicago, Illinois 60612, USA;; 77 Present address: Department of Psychiatry, Kaiser Permanente, San Francisco, California 94118, USA;; 78 Present address: Department of Women's and Children's Health, Center of Neurodevelopmental Disorders (KIND), Karolinska Institutet, 11330 Stockholm, Sweden;; 79 Present address: Department of Psychiatry, University of British Columbia, Vancouver, British Columbia, Canada V6T 2A1;; 80 Present address: Office of the President, Tufts University, Medford, Massachusetts 02155, USA;; 81 Present address: Department of Epidemiology & Biostatistics, Case Western Reserve University, Cleveland, Ohio 44106, USA;; 82 Present address: Hospital for Sick Children, Centre for Addiction and Mental Health, University of Toronto, Toronto, Ontario, Canada M5G1L7

## Abstract

Although multiple reports show that defective genetic networks underlie the aetiology of autism, few have translated into pharmacotherapeutic opportunities. Since drugs compete with endogenous small molecules for protein binding, many successful drugs target large gene families with multiple drug binding sites. Here we search for defective gene family interaction networks (GFINs) in 6,742 patients with the ASDs relative to 12,544 neurologically normal controls, to find potentially druggable genetic targets. We find significant enrichment of structural defects (*P*≤2.40E−09, 1.8-fold enrichment) in the metabotropic glutamate receptor (GRM) GFIN, previously observed to impact attention deficit hyperactivity disorder (ADHD) and schizophrenia. Also, the MXD-MYC-MAX network of genes, previously implicated in cancer, is significantly enriched (*P*≤3.83E−23, 2.5-fold enrichment), as is the calmodulin 1 (CALM1) gene interaction network (*P*≤4.16E−04, 14.4-fold enrichment), which regulates voltage-independent calcium-activated action potentials at the neuronal synapse. We find that multiple defective gene family interactions underlie autism, presenting new translational opportunities to explore for therapeutic interventions.

## Abstract (web summary)

The autism spectrum disorders are complex genetic traits characterized by various neurodevelopmental deficits. Here, the authors analyse defective gene family interaction networks in autism cases and healthy controls and identify potential gene family interactions that may contribute to autism aetiology.

## Graphical abstract

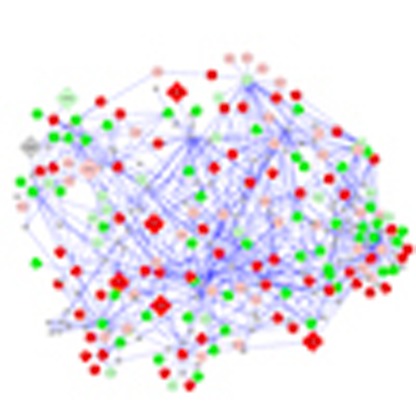

The autism spectrum disorders (ASDs) represent a group of highly heritable childhood neuropsychiatric disorders characterized by a variable phenotypic spectrum of neurodevelopmental deficits of impaired socialization, reduced communication and restricted, repetitive, or stereotyped behaviour^1^. ASDs are four times more common in boys^2^,^3^, and the most recent prevalence estimates across the United States range from 1%^4^ to 2%^5^, although a recent study reported a prevalence as high as 2.6% in a general school-aged population in South Korea^6^. The ASDs have an estimated heritability as high as 90%^7^ based on data on monozygotic twin concordance studies^8^,^9^,^10^, whereas recent estimates of the sibling recurrence risk range from 19% to 22%^11^,^12^.

Despite being highly heritable, the vast majority of family studies suggest that the ASDs do not segregate as a simple Mendelian disorder, but rather display clinical and genetic heterogeneity consistent with a complex trait^13^. Indeed, recent studies estimate that the ASDs may comprise up to 400 distinct genetic and genomic disorders that phenotypically converge^14^,^15^. Common variants such as single-nucleotide polymorphisms seem to contribute to ASD susceptibility, but, taken individually, their effects appear to be small^16^. However, there is increasing evidence that the ASDs can arise from rare or ‘private’ highly penetrant mutations that segregate in families but are less generalizable to the general population^17^,^18^,^19^. Many genes implicated thus far, which are involved in chromatin remodelling, metabolism, mRNA translation and synaptic function, seem to converge in common pathways or genetic networks affecting neuronal and synaptic homeostasis^16^.

Such remarkable phenotypic and genotypic heterogeneity when coupled to the private nature of mutations in the ASDs has hindered identification of new genetic risk factors with therapeutic potential. However, it is noteworthy that many of the rare gene defects implicated in the ASDs belong to gene families. For instance, rare defects impacting multiple members of both the post-synaptic neuroligin (NLGN) gene family^20^ as well as their pre-synaptic neurexin molecular-interacting partners^21^,^22^ have long been reported in patients with ASDs. In addition, a number of other defective gene families with important functional roles have subsequently been well-characterized including ubiquitin conjugation^23^, gamma-aminobutyric acid receptor signalling^24^,^25^,^26^,^27^ and cadherin/protocadherin cell junction proteins^28^ in the brain. Furthermore, multiple defects in voltage-gated calcium channels have been found in schizophrenia^29^, and a defective network of metabotropic glutamate (GRM) receptor signalling was found in both ADHD^30^ and schizophrenia^31^,^32^,^33^,^34^,^35^,^36^, two neuropsychiatric disorders that are highly coincident with the ASDs. Also, the vast majority of significant defective genes identified from recent whole-exome sequences belong to gene families^17^,^18^,^19^.

Many studies have found defective genetic networks in the ASDs^21^,^23^,^37^,^38^,^39^,^40^ (see ref. 16 for review), and we complement these in this work by uncovering new networks and implicating specific defective gene families that may be enriched for novel potential therapeutic targets. Drug-binding sites on proteins usually exist out of functional necessity^33^, and gene families derive from gene duplication events that present additional binding sites for a given drug to exert its effects. Most successful drugs achieve their activity by competing for a binding site on a protein with an endogenous small molecule^41^; therefore, many successful pharmacologic gene targets are within large gene families. Indeed, nearly half of the pharmacologic gene targets fall into just six gene families: G-protein-coupled receptors (GPCRs), serine/threonine and tyrosine protein kinases, zinc metallopeptidases, serine proteases, nuclear hormone receptors and phosphodiesterases^41^. Moreover, many large gene families are localized to pre- and post synaptic neuronal terminals to coordinate the highly complex and evolutionarily conserved process of neurotransmission^42^, which is thought to be compromised to varying degrees in the autistic brain^43^. Therefore, we hypothesize that we may select more druggable targets for the ASDs by enriching for defective interaction networks defined by gene families.

Here we perform a large genome-wide association study (GWAS) of structural variants that disrupt gene family protein interaction networks in patients with autism. We find multiple defective networks in the ASDs, most notably rare copy-number variants (CNVs) in the metabotropic glutamate receptor (mGluR) signalling pathway in 5.8% of patients with the ASDs. Defective mGluR signalling was found in both ADHD^30^ and schizophrenia^31^,^32^,^33^,^34^,^35^,^36^, two common neuropsychiatric disorders that are highly coincident with the ASDs. Furthermore, we find other attractive candidates such as the MAX dimerization protein (MXD) network that is implicated in cancer, and a Calmodulin 1 (CALM1) gene interaction network that is active in neuronal tissues. The numerous defective gene family interactions we find to underlie autism present many novel translational opportunities to explore for therapeutic interventions.

## Results

To identify and comprehensively characterize defective genetic networks underlying the ASDs, we performed a large-scale genome association study for copy-number variation (CNVs) enriched in patients with autism. By combining the affected cases from previously published large ASD studies^21^,^23^,^28^,^44^ with more recently recruited cases from the Children’s Hospital of Philadelphia, we executed one of the largest searches for rare pathogenic CNVs in ASDs to date. In sum, 6,742 genotyped samples from patients with the ASDs were compared with those from 12,544 neurologically normal controls recruited at The Children’s Hospital of Philadelphia (CHOP).

These cases were each screened by neurodevelopmental specialists to exclude patients with known syndromic causes for autism. Genotyping was performed at CHOP for the vast majority of the ASD cases as well as all the controls. After cleaning the data to remove sample duplicates and performing standard QC for CNVs, we first inferred the continental ancestry of 5,627 affected cases and 9,644 disease-free controls using a training set defined by populations from HapMap 3 (ref. 45) and the Human Genome Diversity Panel^46^ (Table 1). Using this QC criteria, we estimated that the sensitivity and specificity of calling CNVs is ~\n70% and 100%, respectively, across 121 different genomic regions assayed by PCR (Methods). Across all ethnicities, there was an increased burden of CNVs in cases versus controls, a statistically significantly difference (*P*≤0.001) in the larger European (63.3 versus 54.5 Kb, respectively) and African-derived (70.4 versus 48.0 Kb, respectively) populations.

**Figure 1:**
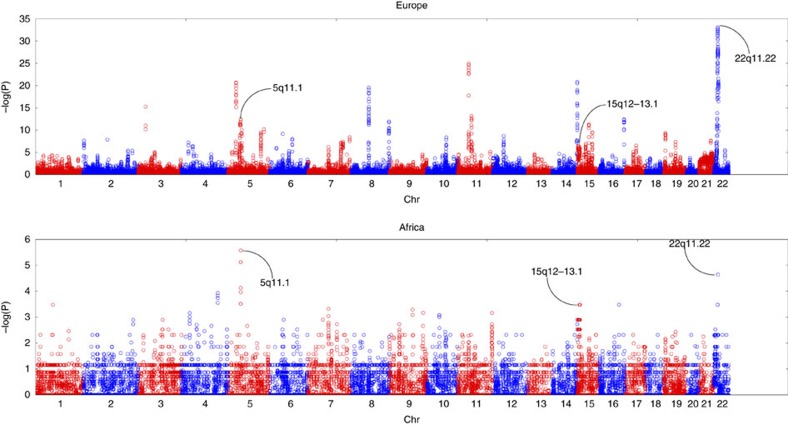
Significance of CNVRs by GWAS of ASDs in European-derived or African-derived populations. The Manhattan plots show the −log10 transformed *P*-value of association for each CNVR along the genome. Adjacent chromosomes are shown in alternating red and blue colours. The regions discovered in Europeans (*P*≤0.0001) that replicated in Africans (*P*≤0.001) are highlighted with black arrows labelled by chromosome band. GWAS of 4,634 cases versus 4,726 controls in Europeans is shown on top and GWAS of 312 cases versus 4,173 controls in Africans is shown below.

We then searched for pan-ethnic CNV regions (CNVRs) discovered in the European-derived data set (4,602 cases versus 4,722 controls; *P*≤0.0001 by Fisher’s exact test) and replicated in an independent ASD data set of African ancestry (312 cases versus 4,169 controls; *P*≤0.001 by Fisher’s exact test) with subsequent measurement of overall significance across the entire multi-ethnic discovery cohort (5,627 cases versus 9,644 controls) for maximal power (Fig. 1, Table 2). On the basis of these selection criteria, two large well-known ASD risk loci emerged that harboured multiple duplications in the Prader Willi/Angelman syndrome (15q11–13) critical region, and multiple deletions were detected in the DiGeorge syndrome (22q11) critical region, albeit notably smaller than the 22q11 deletion syndrome. A third locus harbouring deletions in poly ADP-ribose polymerase family 8 (PARP8) on chromosome 5q11 was also discovered. PARP8 was previously identified as associated with the ASDs in a Dutch population^47^, but it has not previously been described for its pan ethnic distribution across European-derived and African-derived populations.

We examined the genetic interaction networks derived from gene families with members localized to the the Prader Willi/Angelman syndrome (15q11-13) critical region, the DiGeorge syndrome (22q11) critical region, and the novel PARP8 (5q11) region using a method previously applied to ADHD^30^; however, hardly any of the most significant genes harbouring significant CNVRs clustered within gene families. Consequently, we broadened our search for gene family interaction networks (GFINs) and searched the entire genome for GFINs with CNVs enriched in autism. For every gene family, we defined a GFIN as the genetic interaction network spawned by its multiple duplicated members. We used standard HUGO^48^ gene names to define 1,732 GFINs across which we searched for enrichment of network defects associated with the ASDs. However, because there is an *a priori* excess of CNV burden in ASD cases over disease-free controls (Table 1), larger GFINs are expected to display significant enrichment of case defects by virtue solely of their increased size and complexity. Therefore, for each GFIN, we used a network permutation test of case enrichment across 1,000 random sets of networked genes to control for the GFIN size and complexity. With this approach, we robustly identified network defects associated with the ASDs by minimizing statistical artefact derived from any *a priori* excessive CNV burden in cases over controls, as well as other unknown biases that may be inherent in the human interactome data^49^,^50^,^51^ that we mined.

**Figure 2:**
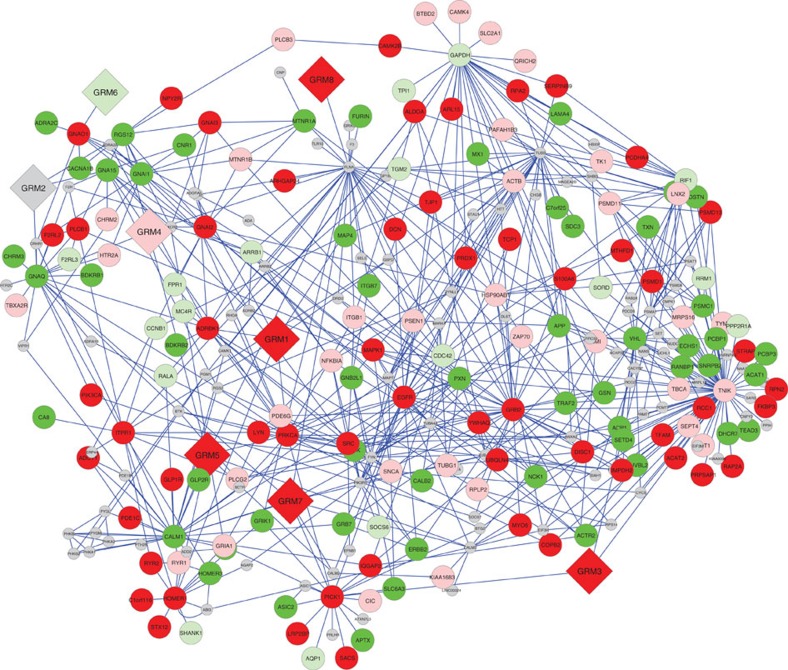
Enrichment of optimal CNVRs across mGluR network of genes. Nodes of the network are labelled with their gene names, with red and green representing deletions and duplications, respectively, while grey nodes lack CNV data. Dark and light colours represent enrichment in cases and controls, respectively. The genes defining the network are shown as diamonds, while all other genes are shown as circles. Blue lines indicate evidence of interaction.

Out of 1,732 GFINs, we used the network permutation test to rank 1,557 GFINs with defined CNVs for enrichment of genetic defects in the ASDs. Among the top GFINs (Table 3) was the metabotropic glutamate receptor (mGluR) pathway defined by the GRM family of genes that impacts glutamatergic neurotransmission. The GRM family contains eight members, all of which were defined in the human interactome to cumulatively spawn a GFIN of 279 genes (Fig. 2). Across this GFIN for the GRM family of genes, we found CNV defects in 5.8% of European-derived ASD cases (265/4,602) versus only 3% of ethnically matched controls (153/4,722), a 1.8-fold enrichment of frequency (*P*_Fisher_ ≤2.40E−09). By 1,000 random network permutations, we found this excess of enrichment across cases in the mGluR pathway to also be statistically significant (*P*_perm_ ≤0.05). In addition, 69.2% (124/181) of the informative genes within our mGluR network showed an excess of CNVs among cases. However, the component genes that harbour the most significant CNVRs contributing to this overall network significance reveal that the duplicated mGluR genes themselves (GRM1, GRM3, GRM4, GRM5, GRM6, GRM7 and GRM8) fail to achieve significance individually, although there is a trend for an excess of CNV defects across a specific subset of mGluR receptors (GRM1, GRM3, GRM5, GRM7, GRM8) that is unique to cases (Supplementary Table 1).

Many large studies of CNVs implicate genes within the glutamatergic signaling pathway in the aetiology of the ASDs^21^,^23^,^37^,^38^,^39^,^40^, and SNP^52^,^53^ and CNV duplications^54^ of GRM8 have been reported in association with the ASDs before in humans. Moreover, a recent functional study demonstrated that in mouse models of tuberous sclerosis and fragile X, two different forms of syndromic autism, the autistic phenotype was ameliorated by modulation of GRM5 in opposite directions for each syndrome, which suggests that GRM5 functional activity is central in defining the axis of synaptopathophysiology in syndromic autism^55^. Our GRM network findings implicate rare defects in mGluR signalling also contribute to the ASDs outside of fragile X and tuberous sclerosis, and we posit that functional mGluR synaptopathophysiology may be initiated from many dozens if not hundreds of defective genes within the mGluR pathway that may account for as much as 6% of the endophenotypes of the ASDs (Table 3).

In addition, we recently demonstrated the importance of mGluRs in ADHD^30^,^56^, a highly co-incident neuropsychiatric disorder within the autism spectrum. However, in contrast to ADHD where defects within the mGluR receptors themselves (GRMs) were among the most significant copy-number defects contributing to the overall network significance, we found that in the ASDs defects of component GRMs contributed only modestly to the overall significance of the mGluR pathway. Nonetheless, the defects within GRM1, GRM3, GRM5, GRM7 and GRM8 that we identified as unique to cases and thus enriched are the same GRMs we identified as being pathogenic in ADHD and may impact glutamatergic signalling.

Among the most highly ranked GFINs by permutation testing, the MAX dimerization protein (MXD) GFIN (P_Fisher_ ≤3.83E−23, enrichment=2.53, *P*_perm_ ≤0.042) was the most enriched. The MXD family of genes encode proteins that interact with MYC/MAX network of basic helix-loop-helix leucine zipper (bHLHZ) transcription factors that regulate cell proliferation, differentiation and apoptosis (MIM 600021)^57^; MXD genes are important candidate tumour suppressor genes as the MXD-MYC-MAX network is dysregulated in various types of cancer^58^. Interestingly an epidemiological link between autism and specific types of cancer has been reported^59^, and anticancer therapeutics were recently shown to modulate ASD phenotypes in the mouse through regulation of synaptic NLGN protein levels^60^. Within the component genes contributing to the MXD GFIN significance, duplications in PARP10 (*P*≤4.06E−11, OR=2.04) and UBE3A (1.50E−06, OR=inf) are the most significantly enriched (Supplementary Table 2). It is notable that we found PARP8 as significant across ethnicities as described earlier (Table 2), and we previously described the importance of structural defects in UBE3A in the ASDs^23^.

Other notable significant GFINs uncovered were POU class 5 homeobox (POU5F) GIFN (*P*_Fisher_≤2.96E−17, enrichment=2.3, *P*_perm_ ≤0.008, and the SWI/SNF related, matrix associated, actin-dependent regulator of chromatin, subfamily c (SMARCC) GFIN (*P*_Fisher_ ≤1.22E−09, enrichment=1.9, *P*_perm_ ≤0.035). The POU5F family of genes encodes for transcription factors containing a POU homeodomain, and their role has been demonstrated in embryonic development, especially during early embryogenesis, and it is necessary for embryonic stem cell pluripotency. Component genes of the SMARCC gene family are members of the SWI/SNF family of proteins, whose members display helicase and ATPase activities and which are thought to regulate transcription of certain genes by altering the chromatin structure around those genes. Most interestingly, the KIAA family of genes ranked among the top GFINs (*P*_Fisher_ ≤3.12E−23, enrichment=1.6, *P*_perm_ ≤0.040). KIAA genes have been identified in the Kazusa cDNA sequencing project^61^ and are predicted from novel large human cDNAs; however, they have no known function.

**Figure 3:**
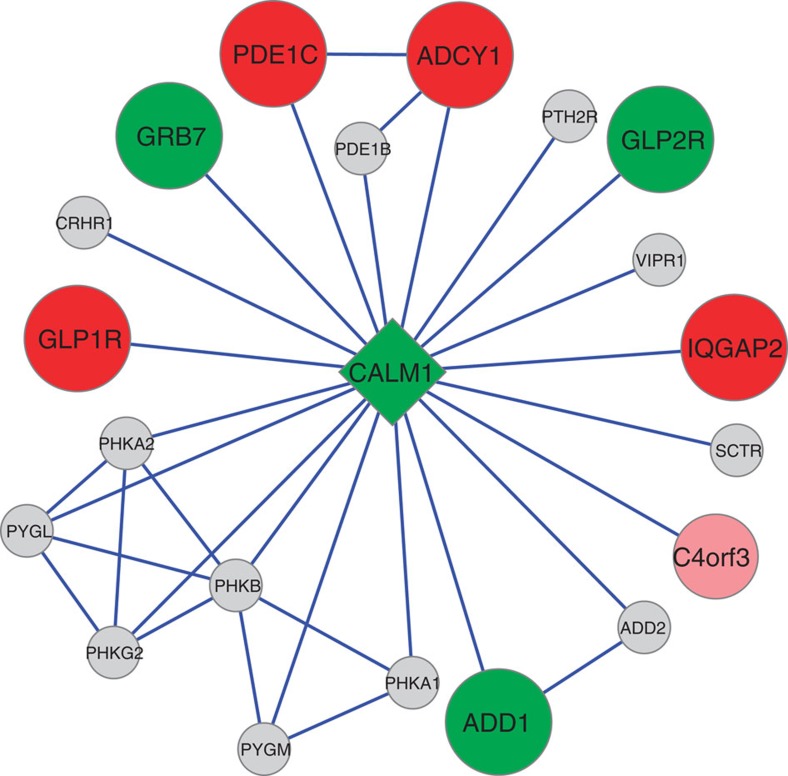
Enrichment of optimal CNVRs across CALM1 network. The first degree-directed interaction network defined by CALM1 is shown.

We also hypothesized that some component members of gene families may contribute disproportionately to the significance of a GFIN because they are highly connected to interacting gene partners that are enriched for CNV defects in ASD. Therefore, we decomposed the 1,732 gene families into their 15,352 component duplicated genes of which 1,218 had defined networks with data to test for significance by genome-wide network permutation. The calmodulin 1 (CALM1) gene interaction network ranked highest by network permutation testing of case enrichment for CNV defects across 1,000 random gene networks (Fig. 3, Table 4) and represents a novel and attractive candidate gene for the ASDs. Across the CALM1 network, we found CNV defects in 14/4,618 cases versus only 1/4726 controls (*P*_fisher_ ≤4.16E−04, enrichment=14.37, *P*_perm_ ≤0.002), and these defects were distributed such that 90% (9/10) of genes that harboured CNVs in the CALM1 interactome were enriched in cases. Closer inspection of the most significant CNVR contributing to the CALM1 network significance (Supplementary Table 3) revealed that no single gene was significant on its own; instead, with the exception of only one gene (PTH2R), each contributing CNVR tagged highly penetrant rare defects unique to cases. Calmodulin is the archetype of the family of calcium-modulated proteins of which nearly 20 members have been found. Calmodulin contains 149 amino acids that define four calcium-binding domains used for Ca^2+^-mediated coordination of a large number of enzymes, ion channels and other proteins including kinases and phosphatases; its functions include roles in growth and cell cycle regulation as well as in signal transduction and the synthesis and release of neurotransmitters [MIM 114180]^57^.

Among other highly ranked first degree gene interaction networks were the nuclear receptor co-repressor 1 (NCOR1; *P*_fisher_ ≤1.11E−06, enrichment=13.37, *P*_perm_ ≤0.004) and BCL2-associated athanogene 1 (BAG1; *P*_fisher_ ≤2.18E−04, enrichment=15.40, *P*_perm_ ≤0.014) networks. NCOR1 is a transcriptional coregulatory protein that appears to assist nuclear receptors in the downregulation of DNA expression through recruitment of histone deacetylases to DNA promoter regions; it is a principal regulator in neural stem cells^51^. The oncogene BCL2 is a membrane protein that blocks the apoptosis pathway, and BAG1 forms a BCL2-associated athanogene and represents a link between growth factor receptors and antiapoptotic mechanisms. The *BAG1* gene has been implicated in age-related neurodegenerative diseases, including Alzheimer’s disease^62^,^63^.

In summary, given the private nature of mutations in the ASDs, considering the cumulative contributions of rare highly penetrant genetic defects boosts our power to discover and prioritize significant pathway defects. As a result, our comprehensive, unbiased analytical approach has identified a diverse set of specific defective biological pathways that contribute to the underlying aetiology of the ASDs. Among GFINs robustly enriched for structural defects, the most enriched was that of the MXD family of genes that has been implicated in cancer pathogenesis^58^, thereby providing concrete genetic defects to explore the reported coincidence of specific cancers with the ASDs^59^. The most highly ranked component duplicated gene interaction network involves defects in CALM1 and its multiple interacting partners that are important in regulating voltage-independent calcium-activated action potentials at the neuronal synapse. Moreover, we found significant enrichment for defects within the GFIN for GRM that defines the mGluR pathway that has previously been shown to be defective in other neuropsychiatric diseases^29^,^30^. While specific mGluR gene family members have been shown to underlie syndromic ASDs^55^, our findings suggest that rare defects in mGluR signalling also contribute to idiopathic autism across the entire GFIN for *GRM* genes.

Consequently, in addition to specific neuronal pathways that are expected to be defective in the ASDs like those defined by GRM and CALM duplicate genes, we implicate completely novel biological pathways such as the MXD pathway specific forms of which may be associated with the ASDs^59^. Given the unmet need for better treatment for neurodevelopmental diseases^64^, the functionally diverse set of defective genetic interaction networks we report presents attractive genetic biomarkers to consider for targeted therapeutic intervention in ASDs and across the neuropsychiatric disease spectrum.

## Methods

### Ethics statement

The research presented here has been approved by the Children’s Hospital of Philadelphia IRB (CHOP IRB#: IRB 06-004886). Some patients and their families were recruited through CHOP outreach clinics. Written informed consent was obtained from the participants or their parents using IRB approved consent forms prior to enrollment in the project. There was no discrimination against individuals or families who chose not to participate in the study. All data were analysed anonymously and all clinical investigations were conducted according to the principles expressed in the Declaration of Helsinki.

### Sample processing

The majority of cases (5,049 of 6,742) and all controls (12,544) were genotyped with genome-wide coverage using the Infinium II platform across various iterations of the HumanHap BeadChip with 550 K, 610 K, 660 K and 1 M markers by the Center for Applied Genomics at The Children’s Hospital of Philadelphia (CHOP). There were 1,693 cases genotyped by the AGP consortium. All cases and ~\n50% of controls were re-used from previously published large ASD studies^21^,^23^,^28^,^44^. All cases were diagnosed by ADI-R/ADOS and fulfilled standard criteria for ASDs. Duplicate samples were removed by selecting unique samples with the best quality (based on genotyping statistics used to QC samples) from clusters defined by single linkage clustering of all pairs of samples with high pairwise identity by state measures (IBS ≥0.9) across 140 K non-correlated SNPs. Ethnicity of samples was inferred by a supervised k-means classification (*k*=3) of the first 10 eigenvectors estimated by principal component analysis across the same subset of 140 K non-correlated SNPs. We used HapMap 3 (ref. 45) and the Human Genome Diversity Panel^46^ samples with known continental ancestry to train the k-means classifier implemented by the R Language for Statistical Computing^65^.

### CNV inference and association

We called CNVs with the PennCNV algorithm^66^, which combines multiple values, including genotyping fluorescence intensity (Log R Ratio), population frequency of SNP minor alleles (B-allele frequency) and SNP spacing into a hidden Markov model. The term ‘CNV’ represents individual CNV calls, whereas ‘CNVR’ refers to population-level variation shared across subjects. Quality control thresholds for sample inclusion in CNV analysis included a high call rate (call rate ≥95%) across SNPs, low s.d. of normalized intensity (s.d. ≤0.3), low absolute genomic wave artefacts (|GCWF| ≤0.02) and low numbers of CNVs called (#CNVs ≤100). Genome-wide differences in CNV burden, defined as the average span of CNVs, between cases and controls and estimates of significance were computed using PLINK^67^. CNVRs were defined based on the genomic boundaries of individual CNVs, and the significance of the difference in CNVR frequency between cases and controls was evaluated at each CNVR using Fisher’s exact test.

### Gene family interaction networks definition and association

We extended our previous work on ADHD^30^ here to rank all GFINs by a network permutation test. Specifically, using merged human interactome data from three different yeast two hybrid generated data sets^49^,^50^,^51^ accessed through the Human Interactome Database^68^, we defined the directed second-degree gene interaction network for all gene families here just as we did for the sole metabotropic glutamate receptor gene family network in ADHD. Specifically, here we use GFIN to refer to these gene family-derived interaction networks. In sum, we found 2,611 gene families with at least two members based on official HUGO^48^ gene nomenclature, and generated 1,732 GFINs using. For 1,557 GFINs with defined CNVs, we calculated an odds ratio of cumulative network enrichment over all genes harbouring CNVs within the network. Moreover, for each GFIN, we quantified its enrichment by a permutation test of 1,000 second-degree gene interaction networks derived from a random set of *N* genes, where *N* is the number of members of a given gene family. Because the CNVs we are focused on are so rare, we are relatively underpowered to achieve significance by permutation testing after correcting for multiple GFIN tests. However, we report all GFINs in the manuscript in order of their nominal/marginal significance.

### Experimental validation of CNVs

Significant CNVRs that we identified were validated using commercially available qPCR Taqman probes run on the ABI GeneAmp 9700 system from Life Technology. Supplementary Data 1 lists 251 reactions that we tested using 121 different genomic probes across 85 different samples for which DNA was available. For deletions, our sensitivity=0.65, specificity=1.00, NPV=1.00 and PPV=0.88. For duplications, our sensitivity=0.68, specificity=0.99, NPV=0.94 and PPV=0.91.

## Author contributions

D.H., Z.W., C.K., J.C., J.G. and H.H. conceived the study. D.H., A.K., K.T., F.M., and H.Q. performed computational analyses. A.M.H., L.V., R.P., and C.K. performed genotyping and experimental validation. H.H. and AGP consortium coordinated sample recruitment. D.H., C.K., Z.W., and H.H. interpreted the results. D.H. and H.H. wrote the manuscript. All authors read, edited and approved the final manuscript

## Additional information

**How to cite this article:** Hadley, D. *et al.* The impact of the metabotropic glutamate receptor and other gene family interaction networks on autism. *Nat. Commun.* 5:4074 doi: 10.1038/ncomms5074 (2014).

## Supplementary Material

Supplementary Tables Supplementary Tables 1-3

Supplementary Data 1 Experimental PCR validation of CNV predictions.

## Figures and Tables

**Table 1:** Distribtion of CNVs across samples and estimated ancestry.

**Continental ancestry**	**Case**	**Control**	**Total**
*Europe*			
Number of samples	4,602	4,722	9,324
*CNV burden (Kb)	63.3	54.5	
			
*Africa*			
Number of samples	312	4,169	4,481
*CNV burden (Kb)	70.4	48.0	
			
*America*			
Number of samples	485	276	761
CNV burden (Kb)	59.1	58.4	
			
*Asia*			
Number of samples	201	350	551
CNV burden (Kb)	56.1	54.1	
			
*Other*			
Number of samples	27	127	154
CNV burden (Kb)	51.5	49.4	
			
*All Ethnicities*			
Number of samples	5,627	9,644	15,271
*CNV burden (Kb)	63.0	51.7	

CNV=copy-number variation. The table shows the distribution of cases, controls and CNV coverage across estimated continental ancestry. For groups of cases and controls across estimated ancestries, the table lists the numbers of subjects that passed quality control and their group-wise CNV burden, defined as the average span of CNVs in Kb for each group.

^*^Statistically significant (*P*≤0.01 by PLINK permutation test) differences in CNV burden are marked with an asterix(*).

**Table 2:** Significant copy-number variable regions.

**CNVR**	**Genes**	**Bands**	**Size (Kb)**	**No. of SNP**	**No. of Case**	**No. of Control**	**All**		**Europe**		**Africa**	
							***P*-value**	**OR**	***P*** **-value**	**OR**	***P*-value**	**OR**
del	ZNF280B	22q11.22	53.4	13	130	0	2.56E−57	Inf	1.94E−33	Inf	3.34E−04	Inf
del	* PARP8	5q11.1	47.7	8	70	8	2.76E−22	15.1	3.84E−13	12.0	2.69E−06	40.9
dup	* GABRB3	15q12	49.0	20	28	0	7.60E−13	Inf	1.50E−06	Inf	3.34E−04	Inf
dup	* GABRG3	15q12	135.3	13	27	1	3.72E−11	Inf	1.60E−05	19.5	3.34E−04	Inf
dup	* HERC2	15q13.1	84.4	2	24	0	4.12E−11	Inf	6.17E−06	Inf	3.34E−04	Inf

CNVR=copy-number variable region; OR=odds ratio. The table shows CNVRs distinguishing cases from controls significant across both European-derived populations (*P*≤0.0001 by Fisher’s exact test) and African-derived populations (*P*≤0.001). For each CNVR, the table lists the type (del or dup), the closest gene impacted, the chromosomal band, the approximate size of the defect (Kb), the number of contributing SNPs, the numbers of affected cases and controls, as well as *P*-value and odds ratio (OR) from Fisher’s exact test for across all populations, and subsets of European-derived and African-derived populations.

^*^Genes with an asterix (*) harbour CNVRs that disrupt their exons of directly, while those without the asterix are located in the genomic region around the intergenic CNVRs.

**Table 3:** Top gene family interaction networks discovered.

**Gene family**		**Enriched genes**		**Cases**		**Controls**		**Gene Network Association**		
**Name**	**Size**	**No.**	**Frequency**	**No.**	**Frequency**	**No.**	**Frequency**	***P***_**fisher**_	**Enrichment**	***P***_**perm**_
BRF	2	242/326	0.742	567	0.123	370	0.078	3.30E−13	1.65	0.040
CCL	24	108/144	0.75	231	0.05	129	0.027	5.62E−09	1.88	0.008
CCNT	2	183/254	0.72	613	0.133	381	0.081	1.10E−16	1.75	0.007
ELAVL	4	108/156	0.692	327	0.071	152	0.032	6.87E−18	2.3	0.043
ERCC	7	263/369	0.713	836	0.182	560	0.119	7.67E−18	1.65	0.035
GRM	8	124/181	0.685	265	0.058	153	0.032	2.40E−09	1.82	0.043
GTF2H	5	152/223	0.682	391	0.085	233	0.049	3.21E−12	1.79	0.049
KIAA	106	268/373	0.718	988	0.215	647	0.137	3.12E−23	1.72	0.045
KPNA	7	256/367	0.698	560	0.122	369	0.078	1.26E−12	1.63	0.028
MXD	3	52/64	0.813	366	0.08	156	0.033	3.83E−23	2.53	0.042
POU5F	2	94/130	0.723	293	0.064	131	0.028	2.96E−17	2.38	0.041
RAD	7	218/309	0.706	535	0.116	339	0.072	9.68E−14	1.7	0.042
SAP	4	111/150	0.74	274	0.06	151	0.032	9.61E−11	1.92	0.040
SMAD	8	845/1,225	0.69	1,782	0.387	1,424	0.302	1.81E−18	1.46	0.039
SMARCC	2	106/147	0.721	239	0.052	131	0.028	1.22E−09	1.92	0.043
SMC	5	88/120	0.733	336	0.073	176	0.037	1.71E−14	2.03	0.034

The table shows significant gene family interaction networks (GFINs) by network permutation testing (*P*_perm_≤0.05) enriched for CNV defects across at least 5% of cases. The table lists the name and size of gene family tested, the number and frequency of network genes enriched in the second degree gene interaction network, the number and frequency of cases harbouring defects across the network, the number and frequency of controls harbouring defects across the network, the significance of association by Fisher’s exact test, the enrichment of CNV defects in cases, and the significance of that enrichment by 1,000 random network permutations.

**Table 4:** Most significant individual gene interaction networks ranked by permutation testing.

**Gene Family Member**	**Enriched Genes**		**Cases**		**Controls**		**Gene Network Association**		
	**No.**	**Frequency**	**No.**	**Frequency**	**#**	**Frequency**	***P***_**fisher**_	**Enrichment**	***P***_**perm**_
AKAP13	7/7	1.00	16	0.0035	1	0.0002	1.14E−04	16.43	0.012
BAG1	7/7	1.00	15	0.0032	1	0.0002	2.18E−04	15.40	0.014
CALM1	9/10	0.90	14	0.0030	1	0.0002	4.16E−04	14.37	0.002
CASP6	16/17	0.94	46	0.0100	6	0.0013	2.96E−09	7.91	0.012
GTF2H3	23/26	0.88	42	0.0091	8	0.0017	3.66E−07	5.41	0.009
MAP3K5	11/12	0.92	34	0.0074	4	0.0008	2.02E−07	8.76	0.012
NCOR1	9/10	0.90	26	0.0056	2	0.0004	1.11E−06	13.37	0.004
PARP1	5/5	1.00	5	0.0011	0	0.0000	2.95E−02	inf	0.012
PTPN13	6/6	1.00	9	0.0019	0	0.0000	1.75E−03	inf	0.007
TCEA1	22/26	0.85	39	0.0084	7	0.0015	5.94E−07	5.74	0.009

The table lists the name and gene family member tested, the number and frequency of network genes enriched, the number and frequency of cases harbouring defects, the number and frequency of controls harbouring defects, and the significance of association by Fisher’s exact test, the odds ratio of the effect size, and the significance of association by random permutation of network while controlling for number of genes tested.
