# Supplementary material for: The impact of the metabotropic glutamate receptor and other gene family interaction networks on autism
Source: Nat Commun. 2014 Jun 13;5:4074. doi: 10.1038/ncomms5074 (PMC4059929; doi:10.1038/ncomms5074)
Supplement: Supplementary Tables — 1-3 [file ncomms5074-s1.pdf]

Supplementary Table 1: Significant CNVRs in the mGluR network. The table shows the 10 most significant CNVRs for 189 genes with data in the GFIN for the GRM gene family across a European-derived population, as well as the most significant CNVRs harbored by the GRM mGluR receptors themselves.

CNV	gene	bands	locus	Size (Kb)	# SNP	# Case	# Ctrl	P	OR
Most significant CNVRs within genes across the mGluR GFIN									
dup	CACNA1B	9q34.3	chr9:140767745-140774721	6.98	2	11	0	4.21E-04	inf
dup	CNR1	6q15	chr6:88858724-88861698	2.98	1	15	8	9.39E-02	1.93
dup	ECHS1	10q26.3	chr10:135174203-135183094	8.89	2	10	0	8.54E-04	inf
dup	HOMER3	19p13.11	chr19:19050957-19054720	3.76	2	9	3	6.68E-02	3.08
del	PSMD1	2q37.1	chr2:232025285-232035793	10.51	1	14	2	1.77E-03	7.20
dup	RANBP1	22q11.21	chr22:20107729-20117344	9.62	1	13	3	9.24E-03	4.46
del	RYR2	1q43	chr1:237273380-237275393	2.01	1	4	0	5.93E-02	inf
del	TJP1	15q13.1	chr15:29812778-30178613	365.84	62	4	0	5.93E-02	inf
dup	TRAF2	9q34.3	chr9:139776401-139821067	44.67	3	6	1	5.83E-02	6.16
dup	TUBA3C	13q12.11	chr13:19743860-19748709	4.85	4	17	8	4.70E-02	2.18
Most significant CNVRs within GRM hubs of mGluR GFIN									
del	GRM1	6q24.3	chr6:146615383-146633818	18.44	3	2	0	2.44E-01	inf
del	GRM3	7q21.12	chr7:86410689-86455535	44.85	9	1	0	4.94E-01	inf
del	GRM4	6p21.31	chr6:33986091-34072561	86.47	26	0	1	1.00E+00	-inf
del	GRM5	11q14.3	chr11:88768276-88841459	73.18	7	4	0	5.96E-02	inf
dup	GRM6	5q35.3	chr5:178313079-178547566	234.49	51	0	2	5.00E-01	-inf
del	GRM7	3p26.1	chr3:7169454-7197715	28.26	11	2	0	2.44E-01	inf
del	GRM8	7q31.33	chr7:126405246-126457778	52.53	11	1	0	4.94E-01	inf

Supplementary Table 2: Most significant CNVRs across genes in the MXD network in European-derived populations. Where large CNVs span multiple genes, the component gene implicated within the MXD gene family interaction network is **bolded**.

CNV	gene(s)	bands	locus	Size (Kb)	# SNP	# Case	# Ctrl	P	OR
dup	SKI	1p36.33	chr1:2204755-2211849	7.10	3	6	1	5.87E-02	6.15
del	SMARCC1	3p21.31	chr3:47749708-47749708	0.00	1	10	2	1.74E-02	5.13
del	HDAC2	6q21	chr6:114260720-114273646	12.93	5	4	0	5.96E-02	inf
del	HDAC9	7p21.1	chr7:18375246-18384157	8.91	2	4	0	5.96E-02	inf
dup	PLEC **PARP10**	8q24.3	chr8:145046663-145059425	12.76	1	243	125	4.06E-11	2.04
del	HDAC7	12q13.11	chr12:48178513-48178604	0.09	2	7	1	3.26E-02	7.17
dup	SNORD115-43 SNORD115-42 **UBE3A** SNORD115-47 SNORD115-45 SNORD115-44 SNORD115-48 SNORD115-36 SNORD109A SNORD115-29 SNORD109B SNORD115-10 SNORD115-11	15q11.2	chr15:25491630-25606727	115.10	11	19	0	1.50E-06	inf
del	**PML** STOML1	15q24.1	chr15:74247944-74343330	95.39	20	6	0	1.45E-02	inf
del	PTPN9 **SIN3A**	15q24.2	chr15:75718670-75806911	88.24	4	7	0	7.19E-03	inf
Most significant CNVRs within MXD hubs of MXD GFIN									
del	SNRNP27 **MXD1**	2p13.3	chr2:70112581-70173629	61.05	10	1	0	4.94E-01	inf
dup	ZFYVE28 **MXD4**	4p16.3	chr4:2256717-2287420	30.70	4	2	0	2.44E-01	inf
del	GPRIN1 RAB24 MIR4281 HK3 UNC5A TSPAN17 SNCB ZNF346 LMAN2 EIF4E1B NSD1 PRELID1 FGFR4 **MXD3** UIMC1	5q35.2 5q35.3	chr5:176024881-176780544	755.66	95	1	0	4.94E-01	inf

Supplementary Table 3: Most significant CNVRs across genes in the CALM1 network in European-derived populations.

CNV	gene	bands	locus	Size (Kb)	# SNP	# Case	# Ctrl	P	OR
del	ADCY1	7p12.3	chr7:45598469-45620959	22491	2	1	0	4.94E-01	inf
dup	ADD1	4p16.3	chr4:2906285-3080173	173889	28	3	0	1.21E-01	inf
del	C4orf3	4q26	chr4:120111190-120399505	288316	31	1	0	4.94E-01	inf
dup	CALM1	14q32.11	chr14:90843792-90868442	24651	4	2	0	2.44E-01	inf
del	GLP1R	6p21.2	chr6:39022698-39055516	32819	23	1	0	4.94E-01	inf
dup	GLP2R	17p13.1	chr17:9708956-9826778	117823	45	1	0	4.94E-01	inf
dup	GRB7	17q12	chr17:37834542-37922259	87718	2	1	0	4.94E-01	inf
del	IQGAP2	5q13.3	chr5:75795407-75805105	9699	4	1	0	4.94E-01	inf
del	PDE1C	7p14.3	chr7:32225283-32228302	3020	5	3	0	1.21E-01	inf
del	PTH2R	2q34	chr2:209280931-209297026	16096	4	0	1	1.00E+00	0
